# Are Intelligent People Better Liars? Relationships between Cognitive Abilities and Credible Lying

**DOI:** 10.3390/jintelligence11040069

**Published:** 2023-04-03

**Authors:** Justyna Sarzyńska-Wawer, Krzysztof Hanusz, Aleksandra Pawlak, Julia Szymanowska, Aleksander Wawer

**Affiliations:** 1 Institute of Psychology, Polish Academy of Sciences, 03-378 Warsaw, Poland; 2 Polish-Japanese Academy of Information Technology, 02-008 Warsaw, Poland; 3 Institute of Cognitive and Behavioral Neuroscience, University of Social Sciences and Humanities, 03-815 Warsaw, Poland; 4 Institute of Computer Sciences, Polish Academy of Sciences, 01-248 Warsaw, Poland

**Keywords:** deception, deception detection, cognitive functions, intelligence

## Abstract

Lying is essential to social communication. Despite years of research, its detection still poses many challenges. This is partly because some individuals are perceived as truthful and reliable, even when lying. However, relatively little is known about these effective liars. In our study, we focused on the cognitive functioning of effective liars. We tested 400 participants who completed tasks measuring executive functions, verbal fluency, and fluid intelligence, and also made four statements (two true and two false, half of them written and half oral). The reliability of the statements was then assessed. Only fluid intelligence was found to be relevant for reliable lying. This relationship was only evident for oral statements, suggesting that the importance of intelligence is highlighted when statements are made spontaneously without prior preparation.

## 1. Introduction

There have been dozens of studies on lying and lie detection over the past few decades. Although much work has been performed to identify behavioral (De Paulo et al. 2003; Ekman and Friesen 1974), verbal (Hauch et al. 2015; Newman 2013), and paraverbal (Adelson 2004; De Paulo et al. 2003; Horvath 1973) indicators of deception, people are still surprisingly poor at detecting lies. A meta-analysis by Bond and DePaulo (2008) conducted on 247 independent samples shows that people’s lie detection accuracy was only slightly higher (54%) than chance. This is partly because effective liars are considered trustworthy, whether telling the truth or lying. However, thus far, little is known about such effective liars. Research to date has focused on such people’s behavior (Sporer and Schwandt 2007; Vrij 2000) and personality traits (Riggio et al. 1988). The ability to lie has been linked to extroversion, honesty–humility, and the so-called dark triad of Machiavellianism, narcissism, and psychopathy, with mixed and often contradictory results (Jonason et al. 2014; O’Connor et al. 2022; Wright et al. 2015). Individuals scoring high on the antisocial personality traits encompassed by the Dark Triad lie more frequently; psychopathy is linked to lying for no reason, narcissism to self-gain lies, and Machiavellianism to white and self-gain lies (Jonason et al. 2014). There is a positive correlation between dark triad scores and perceived deception production ability (Wissing and Reinhard 2019). At the same time, Michels et al. (2020) found that the Dark Triad is not related to lying ability, understood as the ability to lie undetected. Even less is known about the cognitive functioning of effective liars.

Since lying is assumed to be more cognitively demanding than telling the truth, cognitive functions may be crucial for the ability to lie convincingly (Walczyk et al. 2005, 2014). In general, both liars and truth tellers must tell a coherent and plausible story. However, liars cannot rely solely on information from their own memory; they must essentially invent the lie and remember what they have said to accurately recount or reference it later. Research has also shown that most lies are hidden within an otherwise truthful statement (Bond and Speller 2009), so liars will need to monitor and track features of the truth and lie. These suppositions suggest that lying involves several cognitive processes.

We can infer exactly which cognitive functions may play an essential role in the lying process from three different sources: lying theory, neuroimaging research, and the results of experimental studies.

### 1.1. Theories of Lying

Most models of lying, e.g., the Working Memory Model of Deception (Sporer 2016) or the Activation–Decision–Construction Model (ADCM, (Walczyk et al. 2005)) have invoked Baddeley’s (Baddeley and Logie 1999) working memory model, in which information is transferred from long-term memory to an episodic buffer in working memory (WM). According to the ADCM (Walczyk et al. 2005), the decision to produce either response (true or false) is made after activating the exact response in memory. A false response is constructed if a decision is made to lie in the next stage. Inhibitions must then be engaged, allowing real responses to be withheld while being kept in the working memory (WM); this is needed to create a false but possible and credible response. Furthermore, to construct a plausible lie, one must also utilize true information. Moving between true and false response elements requires attention shifting. In summary, lying requires inhibitory control to inhibit the truth when necessary, task-switching to effectively switch back and forth between the truth and the lie, and finally, working memory capacity to manage large amounts of information. The models’ assumptions have been tested in numerous studies.

#### 1.1.1. Working Memory

Evidence for the involvement of working memory in lying and its role in effective lying is provided by studies that show cognitive load increases when lying (Vrij et al. 2017). The tasks used to demonstrate this effect usually involve either recounting events in reverse order or performing a parallel task while lying. Vrij et al. (2017) used a mock crime paradigm and asked participants to recount an event in which they were involved in chronological or reverse order. Police officers who listened to these stories afterward could distinguish between true and false stories in up to 87% of cases. The use of this manipulation significantly improved lie and truth detection and showed that working memory load hinders credible lying. Further, a study by Atkinson (2019) demonstrated that participants who performed better on the Reading Span Task (RSPAN)—which measures working memory capacity—were judged to be more reliable than participants who performed worse on this task. On the other hand, there are also results in which working memory capacity did not correlate with effective lying. In a study by Farrow et al. (2010), people with high working memory capacity took longer to lie than to answer truthfully when compared to those with low working memory capacity.

#### 1.1.2. Task-Switching

Most liars admit to interweaving true and false elements when making up a story (Leins et al. 2013), which requires switching between truth and lies. Research using the Concealed Information Test (CIT, Lykken (1959)) paradigm and tasks based on the instructed lying paradigm has repeatedly shown that there are switch costs for both switching from truth to lie and vice versa, but that responding with the truth is always faster than with a lie (Debey et al. 2015). This means that there are inherent cognitive costs to lying, and the ability to efficiently switch between the true and false aspects of a story can facilitate lying ability. Visu-Petra et al. (2014) showed that task-switching performance was a good predictor of the time taken to lie; those performing better at task-switching took less time to lie. However, studies that did not consider reaction times but assessed the reliability of participant statements revealed that task switching did not affect lying ability (Atkinson 2019).

#### 1.1.3. Inhibitory Control

Evidence of the need for inhibitory control can be found in a study conducted by Debey et al. (2015), who showed that the construction of a lie involves a two-step process in which the first step entails activating a truth upon which a lie response is formulated in the following step. Furthermore, the fact that individuals with greater inhibitory control are better liars is evidenced by research using the Concealed Information Test (CIT, Lykken (1959)) paradigm. Analysis of CIT-obtained reaction times showed that liars with stronger inhibitory control were more similar to truth tellers, with smaller differences in reaction times between event-relevant and irrelevant questions (questions containing similar semantic categories) Debey et al. (2015). However, some studies do not support the role of inhibitory control in plausible lying. For instance, there is no correlation between scores on the go/no-go task Wessel (2018) and the ability to lie Atkinson (2019).

### 1.2. Deception and Executive Functions in Children

The ability to lie is related to children’s cognitive development. Lying requires the engagement of executive functions and is therefore seen by researchers as a sign of children’s cognitive maturity (Talwar and Crossman 2011). Numerous studies indicate that inhibitory control is essential in self-motivated lie-telling Evans and Lee (2011); Talwar and Lee (2008). The ability to tell lies on behalf of others may require cognitive flexibility, understood as switching between telling the truth and lying. The meta-analysis of (Sai et al. 2021) confirmed a positive role of executive functions in children’s lying and development. The publication included 47 papers with 5099 participants aged 2 to 19 years, which yielded 94 effect sizes for executive functions. A statistically significant but relatively small effect was found between children’s lying and executive functioning (r = .13).

### 1.3. Neuroimaging Studies

Using neuroimaging techniques such as fMRI, EEG, and PET, studies have been conducted to assess brain activation patterns in participants when asked to lie or tell the truth. These studies have shown that the ventrolateral and dorsolateral prefrontal cortex is more activated when lying than when telling the truth (Abe 2011; Christ et al. 2009). The ventrolateral prefrontal cortex is the brain area shown to be activated by tasks requiring task-switching ability and inhibitory control. In turn, activation of the dorsolateral prefrontal cortex is typically observed during tasks that require working memory capacity. These studies suggest that executive functions, such as inhibitory control, task-switching ability, and working memory updating, are important for lying.

### 1.4. Intelligence

Intelligence is highly correlated with the cognitive functions described above (Miyake et al. 2001; Salthouse et al. 1998). Its potential role in lying was also emphasized in the Information Manipulation Theory 2 (IMT2, McCornack et al. (2014)). In the IMT2, everyday lies are viewed as quick and easy solutions when honesty could be problematic. The estimated cognitive load of each potential solution is considered when choosing how to respond to a given situation. This implies that people with greater cognitive abilities are more likely to choose an option associated with greater cognitive load because they recognize lying as an option they can easily put into effect. Direct links between intelligence and lying have not yet been thoroughly investigated. According to Vrij et al. (2010), intelligence is linked to eloquence and original thinking, which enables the liar to cope with giving plausible answers in unexpected situations and to think rapidly, which consequently enables them to provide quick and, thus, more convincing answers. Individual research results do not always support either of these suppositions. Michels et al. (2020) conducted a study in which 55 participants were asked to give three statements about personal events, two of which were true and one non-factual. The reliability of these statements was subsequently assessed, and participants’ lying ability was operationalized by the number of raters who failed to identify the non-factual story. The results showed no relationship between intelligence and lying ability. In contrast, people with high intelligence were better at faking personality questionnaire responses to fit the profile desired by a potential employer than people with low intelligence (Pauls and Crost 2005).

### 1.5. The Present Study

In our study, we decided to test whether there is a relationship between fluid intelligence and the ability to lie reliably. Unlike Michels et al. (2020), we did not use subscales from the WAIS-IV (verbal comprehension, perceptual reasoning, working memory, and processing speed) but Raven’s Advanced Progressive Matrices (RAPM) to measure intelligence. We also examined a much larger group of participants and focused on lying in a situation where one has to produce a longer narrative about a general topic in a paradigm where lying ability is measured not by the number of errors made or reaction time, but by whether others notice the lie. However, above all, we looked at two types of lies: oral and written. In writing, liars have more time to plan their utterances and can edit them. In some studies where only written statements were analyzed, this affected—among other things—their length: participants used more words when lying than when they were telling the truth (in the case of oral statements we observe the opposite effect—lies are shorter) (Hancock and Dunham 2001). Such characteristics may influence whether a given utterance is perceived as true. Notably, in our study, participants had no time to prepare oral statements; they made them as soon as they learned the provided topics. We believe that this lack of lying preparation time may be crucial in detecting differences between high and low RAPM performers.

We also measured the working memory, task switching, and inhibitory control of our participants. Following Vrij et al. (2010) suggestions about the role of eloquence in the lying process, we also used verbal fluency tasks.

We also conducted linguistic analyses of the differences between credible and non-credible statements (rated as true and false). To date, much research has been performed on the properties of true and false statements. In contrast, little attention has been paid to the properties of statements that cause them to be rated as true, regardless of their actual veracity.

We assumed that those participants considered more reliable (i.e., those whose lies went undetected) would have higher scores on tasks measuring all cognitive functions and the intelligence test. These differences would be more pronounced in oral statements.

## 2. Method

### 2.1. Participants

A total of 400 subjects aged 18–60 (F = 226; Mage = 30.58, SD = 9.63) took part in this study. Overall, 4.5% of participants completed elementary school, 46.5% had a high school education, and 49% had a college education. Their native tongue was Polish. Participants were recruited through social media and online job posting sites. Each participant received a financial reward of 100 PLN (approximately 25 EUR) for participating in this study. Ethical approval for this study was obtained from the relevant ethics committee. All participants gave their informed consent in writing and were informed that they had the right to withdraw from this study at any time.

### 2.2. Procedure

#### 2.2.1. Statements

This study was conducted in a laboratory at the Institute of Psychology of the Polish Academy of Sciences. In the first stage, participants completed a short questionnaire in which they marked their views on twelve topics known to polarize public opinion. Topics included various social, political, economic, and sports issues (for the full list, see Table 1). Two topics were selected on which the participants had a clear position. Participants were then asked to generate four statements. Two of these were related to a single topic and were spoken orally and recorded. The other two were typed on an online form. One statement on a topic was consistent with the actual position of the participant, while the other presented an opposite point of view. In total, we collected 1600 statements using this method. There were four statements from each participant: two true (one oral and one written) and two false (again, one oral and one written). Participants spoke for at least two minutes during the oral speech and had at least five minutes to create the written statement; they were asked to state their position on each statement and justify it, and were also encouraged to provide both their subjective opinion and arguments on selected topics, which in turn could include both verifiable facts and unverifiable experiences and feelings. Participants were informed about the aim of this study and were told that other people would be judging their statements and trying to guess their true views, so they should be as convincing and credible as possible when making both statements. The order of the statements, both in terms of message type and truthfulness/falsehood, was randomized.

#### 2.2.2. Cognitive Evaluation

Following the oral and written statements, participants completed questionnaires and tasks measuring cognitive functioning. The fluid intelligence test was always presented first, while the order of the remaining tasks was randomized. The battery of tasks included the following:

Intelligence

Raven’s Advanced Progressive Matrices (*RAPM*; Raven and John (2003)). RAPM contains 48 items, presented as one set of 12 (set I) and another of 36 (set II). Each item presents a pattern matrix in which one element is missing. The task is to select the missing element from a set of given alternatives. Items become increasingly complex as progress is made through each set. The RAPM score was the number of correctly solved matrices in set II.

Verbal fluency

Two tasks tested verbal fluency, in which participants were given one minute to name as many words as possible from a given category (animals) to measure semantic fluency, and that begin with a letter (the letter “p”) to measure phonemic fluency. The verbal fluency index (*VBI*) was the sum of scores for both tasks.

Working memory and inhibition

An N-back (*NB*) task was used to assess the ability to update working memory (Nevin 1969). The stimuli consisted of geometric figures and patterns. Participants were instructed to press a button each time the figure presented on the screen matched the one presented three positions prior in the sequence and to refrain from responding otherwise. The ‘targets’ (lures) presented at the wrong position were considered distractors. Since the stimuli were constantly appearing and disappearing, participants had to update their mental sequence of events. Correct rejection of lures required inhibition. Accuracy measure in the N-back task was operationalized as the difference between hit and miss percentages.

Task switching

A Number–letter task adapted from Rogers and Monsell (1995) was used to measure task switching (*TS*) costs. In the task, a number–letter pair (e.g., 2A) was presented in one of four quadrants on the computer screen. Participants were instructed to indicate whether the number was odd or even when the pair was presented in either of the top two quadrants and to indicate whether the letter was a consonant or a vowel when the number–letter pair was presented in either of the bottom two quadrants. The number–letter pair was presented only in the top two quadrants for the first block of trials, only in the bottom two quadrants for the second block of trials, and in a clockwise rotation around all four quadrants for the third block of trials. Thus, the trials within the first two blocks required no task switching, whereas half of the trials in the third block required participants to shift between these two types of categorization operations. Shift cost has been defined as the difference between the average RTs of the trials that required a mental shift and the average RTs of the trials in which no shift was necessary.

### 2.3. Dataset

#### 2.3.1. Statements

We obtained 1600 statements (800 written statements and 800 transcriptions) from our participants. The automated transcription service “Happy Scribe” was used to transcribe the oral statements. All transcriptions were manually checked and corrected. No changes were made to the written statements. Each transcription and each written utterance was then saved in a separate text file.

#### 2.3.2. Evaluation of the Statements—Veracity Rating

Each statement received three ratings. One came from other participants—at the end of the lab session (after giving their own statements and completing cognitive tasks), participants were given 4 randomly selected statements from other participants to evaluate. Another two evaluations came from 5 raters (F = 3, Mage = 39.4, SD = 12.53) who volunteered to participate in this task. These individuals had no professional preparation and were not involved in lie detection professionally. The role of evaluators was also not performed by people with psychological or legal training. Each of these people evaluated about 600 statements. The judges read the original statement (written) or the transcript (oral) and had to decide whether it was true or not. Thus, the most credible utterance could be scored 3 (all evaluators found it to be true), and the least credible utterance could be scored 0 (no evaluator found the utterance to be true).

### 2.4. Final Dataset

The scores of 318 (written) and 319 (transcriptions) participants were included in the final analyses. We excluded participants whose statements (all or part) were not assessed for veracity. A total of 103 statements were not evaluated, including those of participants who did not understand the instructions (e.g., answered truthfully twice or outright admitted to lying), oral statements that were too short, and written statements consisting of only sentence equivalents. Transcription was not possible for some recordings, or the oral statement was not recorded due to technical problems. Participants whose scores on the computer-based cognitive tasks did not register or who performed the tasks unreliably (too short reaction times or too many errors) were also excluded from the analyses.

### 2.5. Linguistic Analyses

We conducted linguistic analyses to determine what distinguishes credible statements from non-credible statements. We examined variables described in the literature on the differences between true and false statements (Hauch et al. 2015). To measure statement complexity, we used two measures of syntactic complexity—mean dependency distance (MDD) and mean hierarchical distance (MHD)—and one measure of lexical complexity—the Gunning Fog Index (FOG). The number of characters and tokens measured statement length. A dictionary of 5421 positive and negative words in Polish was used to measure the number of positive and negative sentiment words. Concreteness was measured according to the Linguistic Category Model (LCM) theory, in which verbs can be classified into categories according to their degree of abstractness. We used the Polish LCM dictionary (LCM-PL; (Wawer and Sarzyńska 2018)), which contains 6000 of the most common Polish verbs. Finally, we used Spacy-PL to obtain the part-of-speech occurrences. All variables and the methods we used to measure them are described in detail in Sarzyńska-Wawer et al. (2023).

## 3. Results

### 3.1. Accuracy and Truth Bias

We investigated the accuracy of judges in identifying lies and true statements across modalities. The results are presented in Table 2. The chi-square test for the contingency table of modality and statement status revealed no significant association—$\chi^{2}$(1) = 0.88, *p* = .34, showing that judges were equally accurate in identifying truths and lies.

Truth bias refers to the tendency to judge more messages as truths than lies and has been confirmed in numerous studies Levine et al. (1999). In order to assess the truth bias, we used two strategies. First, we assessed the cumulative binomial probability of classifying statements as truth. Secondly, using the Signal Detection Theory (*SDP*) approach, we estimated *c’* (the bias index) to evaluate differences in judgments across modalities. Because our data were gathered across multiple persons and rated by three raters, we estimated truth bias as a ratio of the total number of decisions that statement was truth made by three raters (1874) to the total number of assessments (number of statements multiplied by the number of raters) of truths and lies (3468). The exact binomial test results show no statistically significant difference between the observed number of statements assessed as truths and the expected cumulative probability in the sample of statements (*p* = .999, one-sided). This suggests that the observed number of statements assessed as truths is consistent with the expected probability of success of 0.5, the chance level, in the tested sample.

We calculated separately for the written and transcribed statement *c’* index for correctly recognizing the truth as the sum of correct hits and false alarms (*c* = 0.5 (ZFalseAlarms + ZHits)). We have checked whether the *c’* index differs significantly from 0. The two-sided one-sample *t*-test against 0 showed that there were no statistically significant differences between 0 and the value of the *c’* index for written lies (*t*(288) = 1.12, *p* = .216) or transcribed lies (*t*(288) = 1.18, *p* = .239). In addition, the paired t-test showed no differences between modalities: *t*(288) = 1.82, *p* = .068, and *Cohen’s d* = .14.

### 3.2. The Relationship between Cognitive Measures and Lying

**Figure 1:**
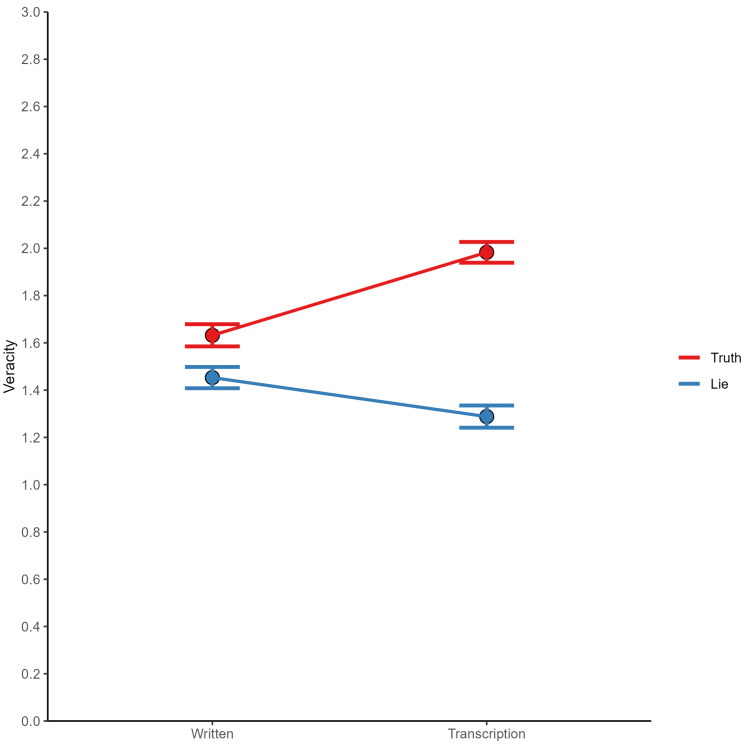
The plot presents the mean scores of statement veracity according to the status and the form of statement. Error bars represent standard error.

Our study focused on veracity ratings, which were measured on a four-point ordinal scale. As this variable represents an ordinal level of measurement, we conducted an ordinal logistic regression analysis to investigate the impact of cognitive measures on lie veracity. Four predictors were used: Raven’s Progressive Matrices score (RAPM), verbal fluency index (VFI), N-back task, and task switching (TS). To account for the substantial interaction between modality and statement status, *F*(1,321) = 61.30, *p* < .001, and $\eta_{G}^{2}$ = .031, we decided to run separate analyses for written and transcribed statements. Our findings revealed that truthful statements were perceived as more credible when they were transcribed than when they were written. Conversely, written statements were rated as more truthful than transcriptions for falsehoods (see Figure 1).

In each case, we first built a model with the RAPM score (Model 1) and subsequently added VFI (Model 2), NB (Model 3), and TS (Model 4). The order of introducing predictors into the model reflects our expected strength of the relationship between the predictor and the dependent variable. For oral statements, the results showed that the model with RAPM was better than the null model: $\chi^{2}$(1) = 9.35, *p* = .001. RAPM was a significant positive predictor of statement veracity—*W*(1) = 9.07, *p* = .003. Our model explained approximately 3% of variance ($R_{C}^{2}oxSnell$ = .03, $R_{N}^{2}agelkerke$ = .03). Adding the three additional predictors did not improve the model fit, as the models with two, three, and four predictors did not reach significance. As for the written statements, none of the models were significantly better than the null model. All model parameters and comparisons are presented in Table 3.

In summary, the best-fitting model for oral lies contained a single predictor—RAPM. A higher score on the RAPM test was associated with a higher probability of a higher veracity category. In contrast, none of the models for written lies reached significance.

### 3.3. Language Differences between Credible and Non-Credible Statements

The analyses of the statements showed that statements judged true were longer (variables: tokens, characters), more abstract (LCM), more complex in terms of syntax (MDD and MHD) and vocabulary (FOG), and contained fewer positive and negative words (positive sentiment and negative sentiment). We found no differences for personal pronouns or other major parts of speech. Descriptive and test statistics are presented in Table 4 and Table 5.

### 3.4. Model Predictions

We predicted the veracity of each utterance using computer-based classification models based on embeddings to represent texts. In natural language processing, a text embedding is a real-valued vector that encodes the meaning of the text in such a way that texts that are closer in the vector space are expected to be similar in meaning. To represent texts, we used the Universal Sentence Encoder (USE) Yang et al. (2019): a pre-trained multilingual sentence encoding model based on the transformer neural network architecture. It has been shown to be highly effective in tasks such as question answering or information retrieval. The USE supports 16 languages, including Polish, and converts input texts to vectors of 512 real-valued numbers. The vectors are then classified using the Support Vector Machine classifier with a radial kernel, which is a supervised model aimed at predicting text veracity. The selection of this specific solution was motivated by the fact that it was previously identified as the best performer on the task of deception detection, as described in Wawer and Sarzyńska-Wawer (2022). The experiment can be summarized as converting texts to vector form in a way independent of the veracity problem and then predicting veracity from embedding vectors using supervised machine learning. The aim is to examine the potential of automatic veracity recognition methods, especially in comparison with the methods of automatic deception detection.

For predicting veracity in a binary fashion, we converted the target variable as follows: we considered statements with veracity “1” and “2” as not credible, while with veracity “3” and “4” as credible. The results of the 20-fold cross-validation are presented in Table 6. The models did not perform as well as in the case of the deception detection described in Wawer and Sarzyńska-Wawer (2022). Even so, the models were capable of significantly outperforming the most frequent class baselines: for typed utterances, the accuracy of the most frequent class baseline is at 0.52, which was outperformed by the average accuracy of 0.60 achieved by the models (+8%), while for transcribed utterances the baseline accuracy is at 0.58, and was outperformed by the models at 0.65 (+7%).

## 4. Discussion

Of all the cognitive abilities we examined, only fluid intelligence appeared necessary for plausible lying. Its level was positively related to the ability to lie, but this was only evident in spoken statements. No such effect was observed for written statements. We believe this is related to the time that participants had to prepare their statements. For written statements, participants spent more time preparing, editing, and polishing them. As in Michels et al. (2020), this negated the effect of intelligence. On the other hand, intelligence played a greater role when participants had to respond immediately, without preparation or room for revision; those with higher RAPM scores performed better on this task. As with Atkinson (2019), we noted no significant effect of the more basic cognitive functions on reliable lying ability; this may be related to our task, which involved statements about general topics not based solely on personal experience in a laboratory setting where lying detection is not a liability. Previous studies have shown that executive involvement mainly depends on the type of deception (Walczyk et al. 2014). We measured executive functions that may be more important to other kinds or aspects of deception (i.e., high-stakes lies). Above all, the impact of executive functioning might be more apparent in instructed lying paradigms. Alternatively, the tasks we used may measure aspects of cognitive functioning that are unrelated to lying. In addition, we have shown that the models are worse at discriminating between credible and non-credible statements than in the case of deception detection Wawer and Sarzyńska-Wawer (2022), which indicates that veracity assessment is an even more challenging task to tackle using automated means. In our study, the so-called truth bias has not become apparent in raters’ evaluations of statements. The fact that individuals are more likely to ascribe truth to others’ messages than deceit has been confirmed in numerous studies (e.g., Levine et al. (1999); Zuckerman et al. (1984). We assume we did not observe this effect in our study because the raters were familiar with the testing procedure and knew that there were equal numbers of true and false statements. Finally, our analysis of the linguistic differences between well- and poorly identified statements showed that credible statements have similar features (they are longer, more complex, and more abstract) to true statements, which have been analyzed in many previous studies on the linguistic cues of deception (e.g., Hauch et al. (2015); Newman (2013)). These findings are consistent with Levine’s theory of sender behavior and that the style of being and—in this case—properties of a statement relate to its veracity, rather than its truthfulness.

Our results add to previous knowledge on the role of intelligence in lying. We have shown that people who are more intelligent can lie credibly in situations that require a quick response. At the same time, it seems that more complex cognitive functions are important in lies involving the production of a broader narrative, while the role of elementary functions is only evident in paradigms where participants provide simpler responses (CIT, instructed lying).

### Limitations and Further Directions

Our study used a task that is frequently used by lie researchers, in which participants lie about topics that polarize public opinion. This task measures only one of many types of lying, so we need to know to what extent our results can be generalized. Further research should also use more cognitive tasks to accurately measure various aspects of elementary cognitive functions, especially executive functions such as shifting, updating, and inhibition. It seems particularly important to determine the types of lying for which these functions may be important. Of course, as in most studies of lying, how lying in the laboratory differs from lying in everyday life is essential. Creating a paradigm outside the laboratory, where people bear the actual cost of lying and experience the emotions specific to lying, seems crucial to better understanding the processes that underlie lying. Our study focused on the cognitive abilities that characterise effective liars. However, in the future, it would be interesting to analyze how traits such as intelligence influence the characteristics of false statements.

In summary, intelligence plays an important role in credible lying in situations where individuals are required to respond immediately to a question without any time to prepare or revise their statements. The differences between low and high scorers on the intelligence test were not apparent when the statements were in written form.

## Figures and Tables

**Table 1:** List of topics: (1) Vaccinations should/should not be compulsory; (2) Polish energy should be based mainly on coal/renewable and non-emission sources; (3) People should/should not eat meat; (4) Smartphones and social media positively/negatively affect interpersonal relationships; (5) Abortion should/should not be legal; (6) God exists/does not exist; (7) Robert Lewandowski is/is not the best Polish football player; (8) Jerzy Zięba’s treatments are/are not effective and help people heal/can harm the sick; (9) Poland should/should not accept more immigrants than today; (10) GM food is/is not safe and useful, and we should/should not invest in these kinds of crops; (11) The political situation in Poland is going in the right/wrong direction; (12) In general, most people can/cannot be trusted; and (13) Ewa Chodakowska is/is not the most effective personal trainer in Poland.

	Written		Transcriptions	
Topic	N	%	N	%
1	134	17.6	92	12.5
2	81	10.6	55	7.4
3	76	10	78	10.6
4	54	7.1	55	7.4
5	91	11.9	94	12.7
6	43	5.6	50	6.8
7	60	7.9	53	7.2
8	30	3.9	50	6.8
9	62	8.1	40	5.4
10	34	4.5	34	4.6
11	32	4.2	54	7.3
12	32	4.2	50	6.8
13	30	3.9	31	4.2

**Table 2:** Average accuracy of truth and lie detection.

	Detection Accuracy	
	Written	Transcription
Truth	55%	67%
Lie	50%	56%

**Table 3:** Ordinal logistic regression model parameters and comparisons.

	Written				Transcriptions			
	Model 1	Model 2	Model 3	Model 4	Model 1	Model 2	Model 3	Model 4
Predictors ${}^{a}$								
RAPM	0.03(0.02)	0.03(0.02)*	0.03(0.02)*	0.03(0.02)*	0.05(0.02)**	0.04(0.02)*	0.04(0.02)*	0.04(0.02)*
Fluency		−0.01(0.01)	−0.01(0.01)	−0.01(0.01)		0.01(0.01)	0.01(0.01)	0.01(0.01)
N-back			0.05(0.33)	0.04(0.33)			0.26(0.33)	0.25(0.33)
Switching				0.00(0.00)				0.00(0.00)
Thresholds ${}^{a}$								
0|1	−1.27(0.32)***	−1.59(0.41)***	−1.59(0.41)***	−1.35(0.47)**	−0.60(0.31)*	−0.17(0.39)	−0.19(0.39)	0.05(0.47)
1|2	0.40(0.30)	0.09(0.39)	0.08(0.39)	0.33(0.46)	1.23(0.31)***	1.67(0.40)***	1.66(0.40)***	1.90(0.48)***
2|3	2.89(0.35)***	2.58(0.43)***	2.58(0.43)***	2.83(0.50)***	3.36(0.37)***	3.81(0.45)***	3.80(0.45)***	4.05(0.53)***
Cox and Snell R^2^	0.01	0.01	0.01	0.02	0.03	0.04	0.04	0.04
Nagelkerke R^2^	0.01	0.02	0.02	0.02	0.03	0.04	0.04	0.05
Chi^2 *b*^	3.12	1.6	0.02	1.01	9.36 **	3.09	0.63	0.85
AIC	782.20	782.59	784.57	785.56	793.45	792.36	793.72	794.87
BIC	797.26	801.42	807.16	811.92	808.49	811.17	816.29	821.20
Log Likelihood	−387.10	−386.30	−386.29	−385.78	−392.72	−391.18	−390.86	−390.44
Deviance	774.20	772.59	772.57	771.56	785.45	782.36	781.72	780.87
Num. obs.	319	319	319	319	318	318	318	318

Note: ${}^{a}$ The values represent b coefficient and corresponding SE. ${}^{b}$ The Likelihood ratio tests compare Model 0 to Model 1, Model 1 to Model 2, and Model 3 to Model 4. *** *p* < 0.001; ** *p* < 0.01; * *p* < 0.05.

**Table 4:** Independent Welch t-test for differences between credible and non-credible statements.

Variable	Statistic	df	*p*-Value	Cohen’s d
POSITIVE SENTIMENT_tok	3.318	1129.865	<.001	0.180
NEGATIVE SENTIMENT_tok	2.023	1062.797	0.043	0.110
PERSONAL PRONOUNS3_tok	1.287	1159.535	0.198	0.070
PERSONAL PRONOUNS12_tok	1.252	1115.432	0.211	0.068
MHD	–5.538	1365.188	<.001	–0.294
MDD	–4.845	1273.886	<.001	–0.260
FOG	–1.968	1181.988	0.049	–0.106
LCM_tok	–5.836	1389.608	<.001	–0.301
TOKENS	–12.634	1373.660	<.001	–0.651
CHARACTERS	–12.634	1373.660	<.001	–0.651

*Note*: Variables with the suffix “tok” take into account the length of utterances.

**Table 5:** Group descriptives.

	Non-Credible			Credibile		
	N	Mean	SD	N	Mean	SD
POSITIVE SENTIMENT_tok	612	0.055	0.032	821	0.050	0.025
NEGATIVE SENTIMENT_tok	612	0.027	0.024	821	0.025	0.018
PERSONAL PRONOUNS3_tok	612	0.014	0.014	821	0.013	0.011
PERSONAL PRONOUNS12_tok	612	0.007	0.010	821	0.007	0.008
MHD	612	2.471	0.572	821	2.646	0.617
MDD	612	2.904	0.527	821	3.037	0.498
FOG	612	13.537	6.224	821	14.149	5.243
LCM_tok	612	5.892	8.787	821	9.439	14.129
TOKENS	612	169.877	110.773	821	268.972	184.516
CHARACTERS	612	957.008	583.289	821	1525.447	1005.079

*Note*: Variables with the suffix “tok” take into account the length of utterances.

**Table 6:** Automated recognition of veracity as a text classification problem. Averages of 20-fold cross-validation.

Typed					
veracity	precision	recall	F1	support	accuracy
false	0.58	0.53	0.55	153	**0.60**
true	0.60	0.65	0.63	168	
Transcribed					
veracity	precision	recall	F1	support	accuracy
false	0.69	0.73	0.71	187	**0.65**
true	0.58	0.53	0.56	131	

## Data Availability

Datasets and source code are available at https://github.com/alexwz/deception-intelligence, and full statements (in Polish) at https://github.com/alexwz/deception-analyses.
